# Effects of Highland Barley Flour with Different Particle Sizes on the Characteristics of Reconstituted Flour and Noodles

**DOI:** 10.3390/foods12051074

**Published:** 2023-03-02

**Authors:** Haibo Liu, Jiaojiao Duan, Jing Zhu, Xiong Liu

**Affiliations:** 1 College of Food Science, Southwest University, Chongqing 400715, China; 2 College of Food Science, XinYang Agriculture and Forestry University, Xinyang 464000, China

**Keywords:** particle size, flour characteristics, highland barley noodles, cooking properties

## Abstract

To study the effects of highland barley flour with different particle sizes on dough characteristics and noodle quality, highland barley flours (median particle sizes of 223.25, 143.12, 90.73, 42.33 and 19.26 μm, respectively) were mixed with the wheat flour to make noodles. The damaged starch content of highland barley flour with five particle sizes was 47.0, 61.0, 62.3, 102.0, and 108.0 g/kg, respectively. The reconstituted flour containing highland barley powder with smaller particle sizes showed higher viscosity and water absorption. The smaller the particle size of barley flour, the lower the cooking yield, shear force and pasting enthalpy of the noodles, and the higher the hardness of the noodles. As the particle size of barley flour decreases, the structural density of the noodles increases. This study is expected to provide a constructive reference for the development of barley-wheat composite flour and the production of barley-wheat noodles.

## 1. Introduction

The noodles were one of the traditional Chinese dishes, and the main ingredients are wheat flour, water and salt. Meanwhile, noodles have a long history in East Asia and are one of the most important staple foods in China and other Asian countries. Among them, China makes up about 20–50% of the overall consumption of wheat flour in Asia [1,2]. In order to meet people’s needs for a diversified and healthy diet, various non-wheat noodles made from whole-grain ingredients have been mass-produced and widely consumed [3]. For instance, noodles made with highland barley, sweet potatoes, brown rice, buckwheat, and peas are more popular with consumers than wheat flour in China [4]. Among them, highland barley is a food crop with particularly great nutritional value. Highland barley has a high content of dietary fiber, protein, minerals, and amino acids. In addition, it also contains a lot of bioactive substances (flavonoids, polyphenols and, β-glucan) [5], which have physiological effects such as lowering cholesterol, lowering blood sugar, and regulating immunity [6,7,8]. Therefore, the development and application of barley in noodle products has a broad prospect. However, the gluten protein content in barley is low, and it is not easy to process and shape. Thus, it is usually made into noodles using wheat flour or gluten protein mixed with it. Flour components (such as starch, gluten, and dietary fiber) can significantly contribute to differences in noodle quality. These components have significant differences in molecular structure, particle size, and thermal properties, which can significantly affect the properties of noodles [9,10].

Particle size, a critical parameter for measuring the flour characteristics, significantly affected the characteristics of dough and the product quality of the flour [11]. These observations and conclusions have already been mentioned in the former studies. Liu et al. [12] reported the effect of whole-wheat flour particle size on the quality attributes of reconstituted flours and tortillas. The results showed that as the particle size of whole wheat flour decreased, the development time of entire wheat flour decreased, while the stabilization time and the degradation time of starch increased. Whole-wheat flour tortilla quality notably improved with a smaller median particle size (130 μm) of whole wheat flour. Qin et al. [13] observed that rice bread made with large particle-size rice flour was smaller compared to the size of rice bread made with small particle-size rice flour. Additionally, the crumb structure was coarser, and the texture of the bread was more complex. It resulted in a less-than-optimal quality of rice bread. Azeem et al. [14] explored the effects of orange-fleshed sweet potato powder (SPF) with different particle sizes (45–355 μm) contributing to the improvement of sweet potato-wheat bread (SPWB). They found that SPWB of SPF with a particle size of 45 μm exhibited a higher specific volume (1.81 mL/g) and a lower hardness (8612.3 N) compared with other bread samples. The particle size distribution has an influence on the characteristics of flour and product. Thus, various types of flour with different particle sizes and product properties should be closely watched and studied.

Previous studies on the particle size of highland barley flour (HBF) concentrated on its impact on the characteristics and nutritional properties of highland barley flour, but few studies examined the characteristics and product quality of reconstituted flour (RF). Therefore, 50% barley flour was used instead of wheat flour (WF) in this study, to investigate the effects of HBF particle size on the characteristics of reconstituted flour and noodles. The results of this study aim to provide references for the production of highland barley flour and highland barley-wheat noodles.

## 2. Materials and Methods

### 2.1. Materials

Highland barley (Zangqing 2000) and Gluten was provided by Changdu Junqin Agricultural and Technology Development Co., Ltd. (Changdu, China). The wheat flour was bought from Weifang Fengzheng Flour Co., Ltd. (Weifang, China). The Rhodamine was purchased from Kolon Chemical Reagent Co., Ltd. (Chengdu, China). All other chemicals and reagents used in the experiments were of analytical grade.

### 2.2. Preparation of Highland Barley Flour (HBF) with Different Particle Sizes

Highland barley kernels were sieved with a sieve to eliminate the impurities, then were milled by a hammer cyclone mill (JXFM110, TOP Instruments Co., Ltd., Zhejiang, China) one, three and six times, and were named HBF-1, HBF-2, and HBF-3, respectively. Next, HBF-3 was further milled with a flow mill (DLF, Dingli Medical Devices Ltd., Wenzhou, China) and named HBF-4, then the HBF-4 was ground for 3 min in an ultra-fine mill (YSC-701, Yanshan Zhengde Machinery Equipment Co., Ltd., Beijing, China) and named HBF-5. Subsequently, the obtained flours were passed through stainless steel sieves with mesh sizes of 40, 60, 80, 100, and 120. Finally, HBF samples were packed in polypropylene bags and stored at 4 °C.

### 2.3. Particle Size and Damaged Starch of HBF

The particle size distribution of HBF was determined with a particle size analyzer (Mastersizer 2000, Malvern, UK) following the method of Li et al. [15] with minor modifications. Enough HBF sample was added to distilled water to achieve the appropriate obscuration factor (10%) and then stirred evenly at 1500 rpm. Parameters to measure HBF particle size distribution include the 10th percentile d (0.1), median d (0.5), 90th percentile d (0.9), and volume-weighted mean D (4,3). The change in volume percentage was recorded in the range of 0.02~2000 μm particle size. The AACCI (76–30.02) method was used to determine the damaged starch content. 

### 2.4. Morphology of Granules

The morphology of HBF granules was observed using the SEM (scanning electron microscope, Phenom-World, Eindhoven, The Netherlands). Briefly, the highland barley powder was evenly fastened to the stage with double-sided adhesive, and gold-plated by ion sputtering, finally a representative image of 5000× was selected. 

### 2.5. Preparation of Reconstituted Flour (RF) and Noodles

An optimum ratio of HBF and WF of 1:1 was obtained through pre-experiments. On account of the lack of gluten protein in highland barley, it was calculated that 7.32 g of gluten flour should be instead of HBF to compensate for the missing gluten protein (9.34% protein content of wheat flour and 63.77% protein content of the gluten flour used in the trial). Thus, each RF sample (100 g) contained 50 g wheat flour, 42.68 g HBF and 7.32 g gluten flour. The RF corresponding to HBF-1, HBF-2, HBF-3, HBF-4, and HBF-5 were named as RF-1, RF-2, RF-3, RF-4, RF-5, respectively.

The optimal ratio of raw materials for noodles was 50 g wheat flour, 42.68 g HBF, 7.32 g gluten flour, 2.4 g salt and 40 g water. Before mixing, 2.4 g of salt dissolved in water. All ingredients were put in a dough mixer (Model NFJ-75, Southstar Machinery Facilities Co., Ltd., Guangzhou, China) and kneaded for three minutes. The dough was then covered with plastic wrap and left at room temperature (25 °C) for 15 min. Afterward, the dough was calendered through a pasta machine (Xingtai Degong Heavy Equipment Manufacturing Factory, Xingtai, China) and sheeted several times using a roll gap setting to obtain smooth dough sheets. Finally, the dough sheets were cut into 20.0 cm long, 2.0 mm wide, and 1.0 mm thick noodles and then dried in a drying chamber (40 °C) for 6 h. The reconstituted flour noodles (RFN) corresponding to RF-1, RF-2, RF-3, RF-4, and RF-5 were named as RFN-1, RFN-2, RFN-3, RFN-4, RFN-5, respectively. In the experiment, noodles made with wheat flour (WFN) served as a control. 

### 2.6. Rapid Viscosity Analysis (RVA) of RF

The pasting viscosities of RF were measured using a Rapid Visco Analyzer (RVA, TCW-3, Newport Science Corp, Melbourne, Australia) according to the method reported by Chen et al. [16] with some modifications. Each RF (3.00 ± 0.01 g) sample and water (25 ± 0.01 g) were placed in an aluminum box and mixed well. The test procedure was as follows: Spin 160 rpm stirring, 50 °C holding for 1 min, 10 °C/min heating to 95 °C, 95 °C holding for 2 min, 10 °C/min cooling to 50 °C, and then 50 °C holding for 2 min.

### 2.7. Mixing Properties of RF

Mixolab (Mixolab-2, Chopin Technologies, Paris, France) was used to measure the mixing characteristics of RF according to the method of Pu et al. [17]. With this device, the torque (Nm), water absorption (WA) (%), development time (DT) (min), and stabilization time (ST) (min) of the dough at different forming stages were determined. The other moments (C_1_, C_2_, C_3_, C_4_, C_5_, Nm) for different periods were deduced from the recorded curves. Starch aging (C_5_-C_4_, Nm) and dough strength weakening (C_1_-C_2_, Nm) were calculated. Calculation of amylase activity (C_3_-C_4_, Nm) and anti-aging effect (C_5_-C_4_, Nm) was conducted.

### 2.8. Color and Cooking Properties of Noodles

The color value (L*, a* and b*) of dried RF noodles was measured by using a chroma meter (UltraScan PRO, HunterLab, Reston, VA, USA). The color difference (ΔE) between WFN and RFN samples was calculated according to the formula: ΔE = [(ΔL*)^2^ + (Δa*)^2^ + (Δb*)^2^]^1/2^. ΔL*, Δa* and Δb* indicate the differences in color values between WFN and RFN samples. The cooking properties of RF noodles were determined by referring to the method reported by Pu et al. [17]. Broken ratio (BR), cooking loss (CL), cooking yield (CY) and optimum cooking time (OCT) for evaluating noodle quality were measured and recorded. Three independent trials were conducted on each noodle sample.

### 2.9. Textural Properties of Noodles

The texture properties of cooked noodles were measured using a TA-XT2i texture analyzer (Stable Micro Systems, Surrey, UK). The noodles were cooked until the white core disappeared, then they were cooled and drained of excess water with filter paper and placed parallel on the measuring table. The calibration was set up with a 500 g load cell and a 15 mm return trigger path. TPA mode parameters [18]: Speed of 1.0 mm/s before, during and after the test, trigger force of 20 g, the compression ratio of 70% using P/36 R probe; shear mode parameters: A/LKB-F probe with pre-test, test and post-test-speeds of 0.8 mm/s, 0.8 mm/s and 2 mm/s, respectively, with 3 g trigger force, 50% compression ratio and 200 p/s data acquisition frequency. Tensile mode parameters: A/SPR probe with a test speed of 3 mm/s and a trigger force of 5 g. The interval height was 30 mm and the data acquisition frequency was 500 p/s. 

### 2.10. Thermal Analysis of Noodles

The thermal properties of the dried noodles were assayed by differential scanning calorimetry (DSC 25 model, TA, New Castle, DE, USA) based on the method of Hemdane et al. [18]. Dry flour was mixed with distilled water in a ratio of 1:3, and the mixture was put into an aluminum pot, then the pot was sealed at room temperature (25 °C) for 24 h. In this way, it ensures that the dry flour fully absorbs the water. The measurement conditions were as follows: Nitrogen flow rate was 20 mL /min and heating rate was 4 °C/min from 20 °C to 120 °C. The starting temperature (To), peak temperature (Tp), concluding temperature (Tc) and enthalpy (ΔH) associated with the pasta flour pasting process were recorded and calculated.

### 2.11. Scanning Electron Microscope (SEM)

The microstructure of the dried noodles was observed using scanning electron microscopy (SEM, Phenom-world, Eindhoven, The Netherlands) using the method of Li et al. [19]. The dried noodles were cut into small pieces and soaked in glutaraldehyde (2.5%, pH 6.8) for 2 h, then rinsing it with phosphate buffer (0.1 M, pH 7.2) and eluted and replaced with ethanol and tert-butanol, finally they were vacuum freeze-dried. Noodle samples were picked and fixed on the stage with double-sided tape for observation. The SEM images were obtained by photographing at 1500× magnification.

### 2.12. Confocal Laser Scanning Microscopy (CLSM)

A confocal laser scanning microscope (CLSM, LSM800, Carl Zeiss, Oberkochen, Germany) was used to analyze the gluten structure of the dried noodles with minor modifications according to the method of Cao et al. [20]. In brief, a sample of dried noodles was cut into small pieces and placed on a slide and stained with 0.025% (*w/w*) Rhodamine B solution for 3 min, then rinsed with distilled water and wiped with filter paper, and images were taken. The excitation wavelength for the test was set to 561 nm. The CLSM images of noodle samples were processed and evaluated using the software “Angio Tool” (National cancer Institute, National Institute of Health, Bethesda, MD, USA).

### 2.13. Statistical Analysis

Three independent tests were carried out for each sample group. Data analysis was processed by SPSS 17.0 (SPSS Inc., Chicago, IL, USA) and final data were presented as mean, standard ± deviation (confidence interval of 0.95) (*p* < 0.05). 

## 3. Results and Discussion

### 3.1. Particle Size Analysis of HBF

The difference in particle size plays a crucial role in the structure, porosity, specific surface area and even functional properties of powdery substances [21]. The HBF particle size distribution analysis is reported in Table 1. The D (4,3) values for HBF-1, HBF-2, HBF-3, HBF-4, and HBF-5 were 282.24, 176.65, 148.08, 80.08, and 25.96 µm. The differences between samples were statistically significant (*p* < 0.05). It is well known that the increase in the grinding intensity will reduce the particle size of the powder granules. The median particle diameters of HBF-1 (223.25 μm), HBF-2 (143.12 μm) and HBF-3 (90.73 μm) declined gradually with increasing the number of milling treatments. However, it was noticeable that the median particle diameters of HBF-4 (42.33 μm) and HBF-5 (19.26 μm) showed a significant decrease due to the different crushing methods. Especially HBF-5 had no particles larger than 150 μm, and the most significant proportion of particles was between 10 and 30 µm. The damaged starch contents of HBF-1, HBF-2, HBF-3, HBF-4, and HBF-5 samples were 47.0, 61.0, 62.3, 102.0, and 108.0 g/kg, respectively. Damaged starch reflected the degree of starch crushing during grain crushing, and its excessive content would adversely affect the dough processing quality [22]. The content of damaged starch gradually increased with the increase in grinding strength. However, the total powder-damaged starch obtained by different crushing methods was quite different, taking HBF-3 and HBF-4 as an example to compare (*p* < 0.05) due to different types of crushing equipment.

### 3.2. Microstructure of HBF

**Figure 1:**
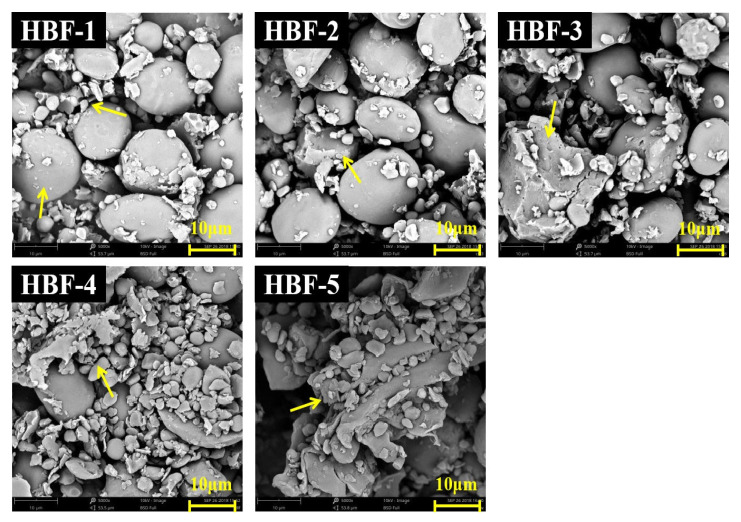
SEM images of highland barley flour with different particle sizes (5000×). HBF-1, HBF-2, HBF-3, HBF-4 and HBF-5 are highland barley flours with median particle size of 223.25, 143.12, 90.73, 42.33 and 19.26 µm, respectively. The yellow arrows in the figure represent highland barley flours with different states.

The microstructure of the HBF is shown in Figure 1. HBF-1 had many intact spherical particles and a few fragments, and they were mainly starch and fiber. As seen from the images of HBF-2 and HBF-3, the starch granules began to collapse and break into small irregular granules. Complete starch granules decreased and fragments increased with the increase in grinding times. This phenomenon was more evident in the images of HBF-4 and HBF-5. The degree of starch granules destruction increased significantly and a state of fragmentation appeared. It may be due to the more substantial mechanical damage effect used to prepare these two particles’ sizes of HBF. Relevant studies have shown that ultrafine pulverization provides high-strength shear, friction, and collision forces during the grinding process, which could effectively reduce the particle size and change its physicochemical properties [23].

### 3.3. Pasting Properties of RF

In most cases, the RVA test is used to better understand the changes in the viscosity of the flour or starch solutions during heating and cooling, as well as to predict the quality of noodles [24,25]. During the process of flour gelatinization, water penetrated the starch crystal structure. It broke the hydrogen bonds between them, and then the starch achieved the transformation from an ordered state to a disordered state. When the flour was heated to a specific temperature, the degree of damage to starch granules increased and the crystal structure disappeared. Finally, the starch expansion granules led to a sharp rise in viscosity, forming a thick paste. 

Table 2 lists the pasting performance of all RF samples. In comparison with the control WF, the RF samples exhibited lower peak viscosity, trough viscosity, final viscosity and setback viscosity due to the difference between the two components. The main components of the WF samples were wheat starch and gluten, at the same time, the RF samples also contained highland barley starch, β-glucans, and some fiber fragments, which affected the gelatinization properties of RF. It could be inferred from the results that the peak viscosity, trough viscosity, breakdown viscosity and final viscosity of RF samples increased significantly with the decrease in HBF particle size (*p* < 0.05). It is probably due to the water absorption and swelling of damaged starch, resulting in the increase in RF viscosity. In addition, as the particle size of highland barley flour decreased, the crystal structure was destroyed, and water penetrated more easily, intensifying the occurrence of pasting [26]. In addition, HBF contained a lot of β-glucans, the β-glucan located in the cell wall of hull-less barley could form a cross-linked network structure in which starch was embedded, resulting in the increased viscosity [27]. The setback viscosity reflected the degree of molecular reordering of starch to form a gel after the starch paste was cooled [28]. This phenomenon indicated that smaller starch and a higher content of damaged starch could obviously enhance the recrystallization ability of RF. The pasting performance is beneficial to better understand the gelatinization and retrogradation of HBF with different particle sizes in RF and can provide a reference for predicting the quality of RFN samples (e.g., Hardness, surface roughness, etc.).

### 3.4. Dough Mixing Properties of RF

The Mixolab test could offer information on dough mixing behaviors. In the test, the torque generated when the dough passed through the two kneading arms was recorded to study the mixing and pasting properties of the flour. Detailed representation of the regular changes measured by Mixolab and the associated parameters were investigated and reported by Rosell et al. [29]. The parameters WA, DT, ST and C_2_ were used to predict the quality of gluten. The C_3_, C_5_ and C_5_-C_4_ parameters stand for the starch gelatinization properties of the RF system as it was heated and cooled [30].

The Mixolab measurement results for different RF samples are shown in Table 3. The experimental results showed that a decreased level of the particle size of HBF from 223.25 μm to 19.26 μm substantially influenced the WA of RF (*p* < 0.05). Compared with the WF group with a WA of 59.4, the WA increased by 12.12%, 16.72%, 17.29%, 20.37% and 21.94% in the HBF-1, HBF-2, HBF-3, HBF-4 and HBF-5 groups, respectively. This was mainly due to the increase in impaired starch with decreasing particle size, leading to the destruction of starch structure and the breakage of molecular starch chains. This resulted in higher exposure of hydrophilic groups in the RF. This conclusion was consistent with that reported by Cai et al. [31]. The DT of WF was 2.62 min, which was significantly shorter than that of RF samples, indicating that coarse bran in HBF disturbed the formation of the gluten network and dough. Subsequently, as the particle size of the HBF decreased, the DT of RF tended to decrease, this result could be attributed to the fact that smaller HBF reduced dough formation resistance. The ST was an indicator describing the strength of the dough. The higher the value, the stronger the dough [1]. Compared with the WF group, the ST of RF was shorter due to components such as fiber and β-glucan in HBF. As the particle size of HBF decreased, the ST of RF showed a trend of decreasing first and then increasing. There were two possible reasons for this phenomenon, and one was that the exposed hydroxyl groups of microfibers bonded to water molecules through hydrogen bonds, which enhanced the stability of the dough [32]. Besides, the continuous refinement of the fibers led to a higher degree of fragmentation, diluting and destroying the structure of the gluten, and reducing the stability of the dough. The C_2_ represents the loss of dough consistency when exposed to both physical-mechanical and thermal stresses [33]. Compared with WF, the C_2_ values of RF were lower and did not significantly (*p* > 0.05) change with the decrease in HBF particle size. During the heating process of the Mixolab test, C_3_ indicated the degree of starch gelatinization and C_3_-C_4_ represented the amylase activity. With the decreasing of HBF particle size, the C_3_-C_4_ value was decreased and the C_5_-C_4_ value was increased. This may be due to the release of powdered amylase from HBF, which increased the activity of starch hydrolysis into small molecules (glucose, maltose, etc.). It further promoted dough fermentation and eventually reduced the starch concentration and pasting viscosity [34]. However, contrary to the report of Wang et al. [33], RF-1 to RF-5, C_5_ and C_5_-C_4_ decreased sequentially, suggesting that the addition of small particles of HBF was less prone to aging, which was beneficial to extend the shelf life of flour products. The discrepancy between the results of these two types of research might arise from the effect of bran size variation or the content of damaged starch on gluten starch regeneration. In this study, the content of damaged starch seemed to play a more vital role than the bran size of HBF.

### 3.5. Color and Cooking Properties of Noodles

Noodles with rich nutrition and bright color were generally more acceptable to consumers. Wheat flour contains yellow pigments such as lutein and carotene. Based on the multiple conjugated double bonds of these compounds, wheat flour was pale yellow in appearance [24]. It could be observed from the results that RFN showed a lower L* value, and higher a* and b* values than those of WFN (Table 4), which is mainly attributed to the lower processing precision of HBF than that of WF. HBF had lower brightness due to richer dietary fiber and ash content. Among RFN samples, the L* values of the other groups were significantly lower than those of RFN-1 and RFN-2 (*p* < 0.05). It may be due to the larger particle size and less damage of HBF-1 and HBF-2, where the pigment component of the bran was not fully exposed. The ΔE values of RFN samples were described as RFN-3 > RFN-5 > RFN-4 > RFN-1 = RFN-2. This shows that the particle size and damage rate of HBF had a greater effect on the color of RFN.

The cooking performance of noodles prepared with different types of RF was measured to better understand the effect of different particle sizes of HBF on the final product quality. Generally speaking, crushing rate, cooking loss, cooking rate, and optimal cooking time were the important criteria for consumers and the industry to evaluate the quality and cooking characteristics of the noodles [17,35]. The results for the cooking characteristics of noodles were also presented in Table 4. From the results, it could be seen that the breakage rate of RFN-1 (14.44%) was remarkably higher than the other groups (0.00%), which is probably due to the fact that HBF-1 with higher roughness reduced the toughness of the noodles. The WFN cooking loss was higher than that of RFN samples, which might be because the fibers in HBF were embedded in the gluten-starch structure to make it more stable, resulting in less starch loss in noodles during cooking. The cooking loss of RFN increased with decreasing particle sizes of HBF, which meant that starch or soluble fiber and other substances with smaller particle sizes were more easily leached and dissolved during the cooking process. Correspondingly, the cooking yield in RNF decreased with the decrease in HBF particle size. The longer optimal cooking time (OCT) of noodles means that the raw materials used to make noodles have higher cooking tolerance. The OCT of WFN was notably shorter than that of other RF groups (*p* < 0.05), and the results suggested that highland barley flour exhibits higher cooking tolerance than wheat flour during cooking.

### 3.6. Textural Characteristics of Noodles 

The textural properties (including TPA, shear, and breaking strength) of the cooked noodles’ components are shown in Table 5. The chewing action of the teeth could be accomplished by the TPA test, which was one of the most generally approbated methods for assessing the sensory properties of cooked noodles [1]. The hardness values of the RFN groups were significantly higher than that of WFN (*p* < 0.05), which probably explained that the fibers in HBF could enhance the hardness of noodles. The hardness of RFN increased with decreasing particle size of HBF, which was in line with the results reported by Chen et al. [36]. Yan et al. [37] found that small starch granules packed between large granules and the gluten network could make the noodles’ structure denser and more compact. The adhesiveness of WFN was significantly higher than that of RFN. This phenomenon was interpreted as wheat flour being easier to form a dense network structure than highland barley flour. The change in adhesiveness values from RFN-1 to RFN-5 suggested that the more significant particle size starch and fibers in highland barley flour had more detrimental effects on the formation of the gluten protein network in the noodles. The springiness of WFN was not significantly different from that of RFN. It was worth mentioning that the chewiness of WFN was similar to that of RFN-5, but they were both lower than other RFN groups, while the resilience of RFN-1 was lower than that of other samples, and the resilience of other samples had no significant difference. Chewiness and resilience values were reported to be highly correlated with the whole gluten network and reflected the viscoelasticity of the dough system [38]. Highland barley flour with a larger particle size (except HBF-5) was evenly embedded in the gluten network, and there were large gaps between macromolecules, resulting in increased chewiness of noodles. However, the large fibers in highland barley flour (such as HBF-1) could entangle the gluten network and reduce the resilience of the noodles. Furthermore, the shearing force of RFN decreased as the particle size of HBF decreased. In contrast, the breaking strength of RFN increased with the decrease in HBF particle size. Meanwhile, the shearing force of RFN-1 and the breaking strength of RFN-5 were remarkably higher than other groups. On the contrary, the breaking strength of RFN-1 and the shearing power of RFN-5 was significantly lower than other groups. This result may indicate that the RFN-1 fibers were hard and coarse as well as lacking in toughness, while the fine fibers of the HBF-5 group had less detrimental effects on the sensory and quality of the noodles. In general, the hardness and breaking strength of RFN-5 were greater than that of WFN, and other characteristics were relatively close to WFN.

### 3.7. Thermal Properties of Noodles

The effect of highland barley flour with different particle sizes on the thermal properties of noodles is shown in Table 6. Values measured by differential scanning calorimetry refer to the thermal stability and gelatinization properties of starch in the noodle samples. It can be seen that RFN samples started to gelatinize at temperatures ranging from 55.43 to 58.54 °C. As the particle size of highland barley flour decreased, it gradually increased and Tc gradually decreased in RFN samples, but all were lower than WFN. Yang et al. [39] found that highland barley flour showed lower To and higher Tc than highland barley starch. This may be caused by the interaction of other components (fiber, β-glucan, etc.) in highland barley flour with starch, affecting gelatinization [40]. The smaller the particle size of barley powder, the more obvious this phenomenon will be due to the uniform distribution of each component and spatial positional resistance. The difference in Tp between the different noodle samples was not significant, indicating that the difference in ingredients did not affect the Tp of the noodles. The H of RFN samples showed a decreasing trend as the particle size of HBF decreased, which was in alignment with the previous report by Asmeda et al. [22]. This could be explained by the fact that intact starch granules (with less damaged starch) must overcome greater resistance and more energy to fully gelatinize.

### 3.8. Microstructure of Noodles

**Figure 2:**
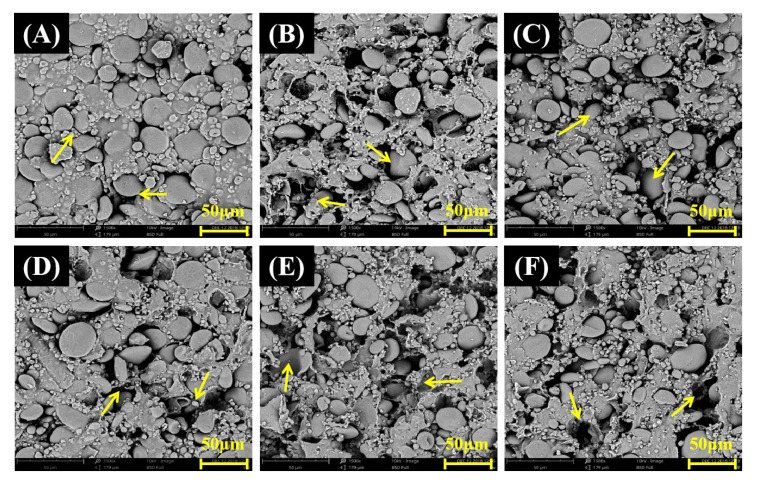
SEM images of the microstructure of noodles made from highland barley flour with different particle sizes (1500×); WFN (**A**), RFN-1 (**B**), RFN-2 (**C**), RFN-3 (**D**), RFN-4 (**E**), RFN-5 (**F**). The yellow arrows in the figure represent the gaps of the internal structure of the noodles.

The microstructure and morphology of the noodles are shown in Figure 2. The SEM image showed that the WFN sample exhibited a smooth and dense structure with many entire starch granules embedded uniformly and regularly in the protein network. However, the RFN samples showed a rough and porous structure with starch granules, fibers and other cellular debris distributed disorderly in the protein network. It was worth noting that the RFN samples contained some broken starch granules. This can be ascribed to the gluten network disruption by the inhomogeneous fibers and starch in the HBF [41]. On the other hand, additional gluten proteins were supplemented in RFN, resulting in uneven distribution and aggregation of the network structure. The structure of RFN-1 was loose, with more and larger pores, fibers and starch granules that were not completely encapsulated in the network. RFN-3 had a denser structure and smaller pores than RFN-2 and RFN-1. Meanwhile, smaller starch granules were observed in RFN-4 and RFN-5, indicating that the finely ground HBF could be reconstituted more uniformly in the wheat flour system, and the starch could be better encapsulated by the gluten protein.

**Figure 3:**
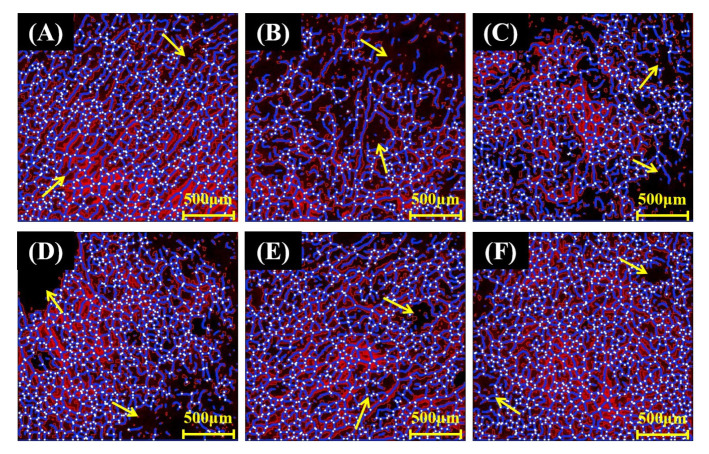
Images of CLSM analysis of noodle gluten network made of highland barley powder with different particle sizes processed by AngioTool software (with junctions shown in white, gluten skeleton shown in purple); WFN (**A**), RFN-1 (**B**), RFN-2 (**C**), RFN-3 (**D**), RFN-4 (**E**), RFN-5 (**F**). The yellow arrows in the figure represent the gaps of gluten.

The CLSM images of noodles samples were processed and quantified with “AngioTool” to obtain seven parameters (Table 7), enabling the quantitative analysis of the gluten network microstructure (Figure 3). Gluten percentage area was the proportion of the gluten network in the selected image of CLSM, reflecting the distribution of gluten in the noodle sample. WFN had the highest gluten percentage area (64.41%), and the gluten percentage area of RFN samples increased with the decrease in HBF particle size. It was worth noting that the gluten percentage area of RFN-5 and WFN (63.09%) had no notable difference (*p* > 0.05). This phenomenon indicated that HBF with larger particle size disrupted gluten stability and uniformity during dough formation, while HBF with smaller particle size was more favorable to the cross-linking of gluten to form a dense network structure. Since bran endpoints were open protein threads, they were often used as a valid reference for measuring network cohesion [42]. The number of gluten endpoints of WFN was 1.60 × 102, which was not significantly different from RFN-4. The gluten endpoints of RFN-1 and RFN-2 were basically the same, but both were higher than that of WFN. The gluten endpoints of RFN samples decreased with the decrease in HBF particle size. The results further illustrated the effect of HBF with different particle sizes on the cross-linking of gluten. Lacunarity was an essential indicator for evaluating the quality of dough [43]. The lacunarity of RFN samples decreased with the decreasing particle size of HBF, and the lacunarity of RFN-5 was close to that of WFN (Table 7). Higher lacunarity values in the gluten network mean that the gluten had more voids of different sizes, which are determined by the amount and size of the components embedded in the gluten. It was shown that there are unevenly distributed holes or gaps of varying sizes in the gluten network of different strengths, embedded with starch granules of different size distributions [43,44,45]. In this study, the difference among RFN noodle samples lies in the component HBF with different particle sizes, which results in different size gaps of gluten. Lacunarity was a property of the gluten network, which could indirectly reflect the cross-linking of gluten protein and the interaction between gluten protein and other components, which provided a new reference for studying the microstructure of the gluten network.

## 4. Conclusions

Differences were found in the physical-chemical properties of barley flour with different particle sizes, including damaged starch content, morphological characteristics and pasting properties. In addition, the particle size of highland barley flour had impacts on the color, cooking properties, textural characteristics, thermal features and internal structure of noodles. It showed that the particle size could be used as an important parameter to describe the characteristics of wheat-highland barley flour and wheat-highland barley noodles. The relevant research results in this study can provide ideas for the development of highland barley noodles with different features, so as to promote the unconventional application of highland barley flour and improve the economic benefits of highland barley. This work explored the effect of whole highland barley flour on the characteristics of reconstituted flour and noodles. However, the main components in highland barley flour (starch, fibers and β-glucan) have different characteristics. The effect of these individual factors on the whole dough system is still unclear, and related studies will be carried out in the future.

## Figures and Tables

**Table 1:** The different particle size distributions and damaged starch content of highland barley flour.

Sample	d (0.1) (μm)	d (0.5) (μm)	d (0.9) (μm)	D (4,3) (μm)	DS (g/kg)
HBF-1	12.79 ± 0.41a	223.25 ± 17.63a	667.88 ± 18.77a	282.24 ± 11.27a	47.0 ± 1.9a
HBF-2	11.71 ± 0.04b	143.12 ± 2.99b	410.18 ± 13.16b	176.65 ± 5.44b	61.0 ± 2.4b
HBF-3	9.99 ± 0.20c	90.73 ± 3.63c	385.21 ± 4.81c	148.08 ± 3.11c	62.3 ± 0.0b
HBF-4	7.04 ± 0.02d	42.33 ± 0.35d	210.17 ± 3.80d	80.05 ± 1.17d	102.0 ± 2.4c
HBF-5	3.57 ± 0.11e	19.26 ± 0.61e	58.00 ± 6.43e	25.96 ± 1.92e	108.0 ± 1.8c

d (0.1), d (0.5), and d (0.9) are the granule sizes at which 10, 50, and 90% of all the particles by volume are smaller, respectively. D (4,3) is the volume-weighted mean diameter; DS, damaged starch. Data are represented as means ± standard deviations, *n* = 3. Values in the same column with different letters are significantly different (*p* < 0.05).

**Table 2:** Effect of highland barley flour with different particle sizes on the pasting property of RF.

Sample	Pv (mPa·s)	Tv (mPa·s)	BD (mPa·s)	Fv (mPa·s)	SB (mPa·s)	PT (min)
WF	2440.00 ± 10.00a	1904.00 ± 29.00a	536.00 ± 19.00c	3182.50 ± 0.50a	1278.50 ± 28.50a	6.37 ± 0.03a
RF-1	1066.67 ± 7.23d	599.33 ± 3.21c	467.33 ± 4.16d	1461.67 ± 8.74c	862.33 ± 6.35c	5.69 ± 0.03c
RF-2	1449.00 ± 46.86c	692.33 ± 18.04b	756.67 ± 34.31b	1656.33 ± 35.23b	964.00 ± 19.08b	5.71 ± 0.08c
RF-3	1489.00 ± 27.18c	708.33 ± 16.26b	780.67 ± 43.41b	1682.67 ± 17.39b	974.33 ± 7.09b	5.78 ± 0.04c
RF-4	1642.00 ± 47.32b	715.33 ± 41.10b	926.67 ± 50.90a	1678.67 ± 52.05b	963.33 ± 25.17b	5.95 ± 0.04b
RF-5	1662.33 ± 15.82b	721.67 ± 24.34b	940.67 ± 11.15a	1676.33 ± 27.79b	954.67 ± 12.66b	6.02 ± 0.04b

Pv, peak viscosity; Tv, trough viscosity; BD, breakdown viscosity; Fv, final viscosity; SB, setback viscosity; PT, peak time. Results are presented as means ± standard deviations (*n* = 3). Values in the same column with different letters are different significantly (*p* < 0.05).

**Table 3:** Effect of highland barley flour with different particle sizes on the Mixolab properties of RF.

Sample	WA (%)	DT (min)	ST (min)	C_2_ (Nm)	C_3_ (Nm)	C_5_ (Nm)	C_3_-C_4_ (Nm)	C_5_-C_4_ (Nm)
WF	59.40 ± 0.36e	2.62 ± 0.18e	10.67 ± 0.15a	0.86 ± 0.03a	2.62 ± 0.01a	2.48 ± 0.02a	1.00 ± 0.01a	0.86 ± 0.07a
RF-1	66.60 ± 0.00d	8.95 ± 0.16a	6.23 ± 0.23d	0.46 ± 0.01b	1.66 ± 0.02b	2.22 ± 0.05b	0.18 ± 0.03d	0.74 ± 0.02b
RF-2	69.33 ± 0.06c	6.07 ± 0.86b	8.40 ± 0.50c	0.47 ± 0.04b	1.51 ± 0.02c	1.98 ± 0.04c	0.24 ± 0.02c	0.71 ± 0.06b
RF-3	69.67 ± 0.06c	5.72 ± 0.47bc	9.20 ± 0.36b	0.47 ± 0.00b	1.50 ± 0.02c	1.88 ± 0.01d	0.29 ± 0.01b	0.67 ± 0.04b
RF-4	71.50 ± 0.61b	4.96 ± 0.12cd	8.93 ± 0.06bc	0.45 ± 0.01b	1.36 ± 0.01d	1.63 ± 0.03e	0.30 ± 0.01b	0.58 ± 0.03c
RF-5	72.43 ± 0.72a	4.57 ± 0.10d	8.87 ± 0.15bc	0.45 ± 0.02b	1.32 ± 0.02e	1.53 ± 0.00f	0.31 ± 0.03b	0.52 ± 0.01c

WA, water absorption; DT, dough development time; ST, stability time; C_2_, weakening of protein network; C_3_, the rate of starch gelatinization; C_5_, the starch retrogradation during the cooling period; C_3_-C_4_, the amylase activity; C_5_-C_4_, the anti-aging effect of the starch. Results are presented as means ± standard deviations (*n* = 3). Means that different small letter superscripts in the same column are significantly different (*p* < 0.05).

**Table 4:** Effect of highland barley flour with different particle sizes on the color and cooking characteristics of noodles.

Sample	L*	a*	b*	ΔE	BR (%)	CL (%)	CY (%)	OCT (min)
WFN	89.56 ± 0.67a	2.09 ± 0.16d	18.13 ± 0.25c	-	0.00 ± 0.00b	6.09 ± 0.07a	153.80 ± 5.34a	1.96 ± 0.04d
RFN-1	73.68 ± 0.43b	5.33 ± 0.11c	19.31 ± 0.20b	16.25 ± 0.14d	14.44 ± 1.93a	4.47 ± 0.16d	105.40 ± 2.10b	3.17 ± 0.00a
RFN-2	73.73 ± 0.41b	5.30 ± 0.23c	18.43 ± 0.50c	16.15 ± 0.12d	0.00 ± 0.00b	4.52 ± 0.29d	103.66 ± 3.83b	2.32 ± 0.17c
RFN-3	69.13 ± 0.11e	5.83 ± 0.06b	21.18 ± 0.23a	20.99 ± 0.21a	0.00 ± 0.00b	4.77 ± 0.08cd	98.09 ± 4.85bc	2.67 ± 0.09b
RFN-4	72.77 ± 0.29c	5.99 ± 0.11ab	20.73 ± 0.30a	17.43 ± 0.18c	0.00 ± 0.00b	4.89 ± 0.17c	97.21 ± 6.83bc	2.44 ± 0.05c
RFN-5	69.92 ± 0.43d	6.19 ± 0.22a	21.11 ± 0.41a	20.28 ± 0.22b	0.00 ± 0.00b	5.25 ± 0.28b	94.28 ± 0.16c	2.61 ± 0.10b

L*, lightness; a*, redness-greenness; b*, yellowness-blueness; BR, broken ratio; CL, cooking loss; CY, cooking yield; OCT, optimal cooking time. “-” indicates the ΔE value of WFN sample is used as control group. Results are presented as means ± standard deviations. Means with different small letter superscripts in the same column are significantly different (*p* < 0.05).

**Table 5:** Effect of highland barley flour with different particle sizes on the texture properties of noodles.

Sample	Ha (N)	Ad (N)	Sp (mm)	Ch (N·mm)	Re	SF (N)	BS (N)
WFN	75.96 ± 0.87e	−0.97 ± 0.09a	0.97 ± 0.02a	51.27 ± 1.98b	0.38 ± 0.03a	2.18 ± 0.03bc	21.24 ± 0.23d
RFN-1	87.88 ± 0.54d	−2.35 ± 0.18d	0.93 ± 0.02a	62.79 ± 2.84a	0.32 ± 0.01b	2.78 ± 0.03a	20.18 ± 0.18e
RFN-2	88.21 ± 0.66d	−1.87 ± 0.12c	0.95 ± 0.03a	62.10 ± 4.69a	0.40 ± 0.02a	2.21 ± 0.03b	23.35 ± 0.42c
RFN-3	89.24 ± 0.48c	−1.46 ± 0.06b	0.96 ± 0.02a	61.89 ± 2.58a	0.39 ± 0.02a	2.20 ± 0.03b	23.82 ± 0.36c
RFN-4	91.24 ± 0.26b	−1.15 ± 0.02a	0.97 ± 0.01a	61.34 ± 0.22a	0.41 ± 0.03a	2.10 ± 0.04c	26.38 ± 0.28b
RFN-5	93.65 ± 0.72a	−1.14 ± 0.09a	0.97 ± 0.03a	57.09 ± 3.66b	0.38 ± 0.02a	1.82 ± 0.02d	28.83 ± 0.31a

Ha, hardness; Ad, adhesiveness; Sp, springiness; Ch, chewiness; Re, resilience; SF, shearing force; BS, breaking strength. Values in the same column with different letters are significantly different (*p* < 0.05).

**Table 6:** Effect of highland barley flour with different particle sizes on the thermal properties of noodles.

Sample	To (°C)	Tp (°C)	Tc (°C)	ΔH (J/g)
WFN	59.05 ± 0.01a	66.40 ± 0.00a	75.43 ± 0.12a	2.14 ± 0.01a
RFN-1	55.43 ± 0.10d	67.40 ± 0.00a	73.58 ± 0.01b	1.95 ± 0.04b
RFN-2	55.31 ± 0.06e	66.79 ± 0.00a	72.27 ± 0.22c	1.72 ± 0.02c
RFN-3	57.76 ± 0.01c	67.92 ± 0.00a	71.51 ± 0.01d	1.37 ± 0.01d
RFN-4	57.70 ± 0.04c	67.31 ± 0.01a	68.39 ± 0.26e	1.14 ± 0.01e
RFN-5	58.54 ± 0.03b	66.67 ± 0.00a	67.21 ± 0.02f	0.99 ± 0.01f

To, onset temperature; Tp, peak temperature; Tc, conclusion temperature; ΔH, gelatinization enthalpy. Values are the mean ± standard deviation. Different letters in the same column are significantly different (*p* < 0.05).

**Table 7:** Quantitative analysis of the gluten network in the different RFN samples determined by AngioTool software.

Sample	GA (×10^4^ μm^2^)	GPA (%)	GJ	JD (×10^−3^)	TGL (×10^3^ μm)	GE (×10^2^)	La (×10^−2^)
WFN	11.13 ± 0.58a	64.41 ± 2.06a	665.5 ± 52.60b	5.9 ± 0.08b	11.85 ± 0.12b	1.60 ± 0.21c	1.68 ± 0.16e
RFN-1	7.75 ± 0.87d	44.84 ± 1.15d	482.5 ± 9.80c	4.4 ± 0.22c	9.51 ± 0.09d	3.28 ± 0.24a	7.76 ± 0.21a
RFN-2	7.97 ± 0.21d	46.10 ± 0.46d	514.8 ± 36.89c	4.6 ± 0.15c	9.67 ± 0.05d	3.16 ± 0.19a	6.79 ± 0.27b
RFN-3	9.23 ± 0.34c	53.39 ± 1.93c	645.1 ± 18.92b	5.8 ± 0.28b	11.04 ± 0.38c	2.70 ± 0.35b	5.73 ± 0.48c
RFN-4	10.03 ± 0.45b	58.02 ± 0.87b	628.7 ± 10.09b	5.61 ± 0.25bc	11.33 ± 0.09c	1.91 ± 0.22c	2.32 ± 0.24d
RFN-5	10.91 ± 0.89a	63.09 ± 0.85a	847.2 ± 50.20a	7.6 ± 0.17a	12.89 ± 0.28a	1.42 ± 0.18d	1.49 ± 0.13e

GA, gluten area; GPA, gluten percentage area; GJ, gluten junctions; JD, junction density; TGL, total gluten length; GE, gluten endpoints; La, lacunarity. Values in the same column with different letters are significantly different (*p* < 0.05).

## Data Availability

Data is contained within the article.
